# Enantiomorphs and taxonomy of three conchological species in flat-shelled snails *Trichocathaica* (Pulmonata, Camaenidae)

**DOI:** 10.3897/zookeys.810.29824

**Published:** 2018-12-20

**Authors:** Barna Páll-Gergely, András unyadi, Takahiro Asami

**Affiliations:** 1 Plant Protection Institute, Centre for Agricultural Research, Hungarian Academy of Sciences, Budapest, Hungary Centre for Agricultural Research, Hungarian Academy of Sciences Budapest Hungary; 2 Adria sétány 10G 2/5., Budapest 1148, Hungary Unaffiliated Budapest Hungary; 3 Department of Biology, Shinshu University, Matsumoto 390-8621, Japan Shinshu University Matsumoto Japan

**Keywords:** Chirality, enantiomorphism, Gastropoda, left-right reversal, penial morphology, Stylommatophora

## Abstract

Biomodal (flat/globular or slender/tall) shell/body shapes are associated with dichotomous (simultaneous reciprocal or non-reciprocal) modes of copulation behaviour in the fully-shelled stylommatophoran snails. In flat-shelled groups that copulate simultaneously reciprocally, no study has found an example of enantiomorphism that persists within a population. However, the original description of a flat camaenid snail, *Trichocathaicaamphidroma*, noted that it is dextral- or sinistral-coiled. By examination of shell surface morphology, we found that shell specimens classified as those of this species include shells of three different morphological species. Namely, *T.amphidroma*, *Trichocathaicavestita* (Pilsbry, 1934), **comb. n.**, and *Trichocathaicamacrosquamata* Páll-Gergely, **sp. n.** In each of the three species, both sinistral and dextral shells have been collected from presumably one area. Ethanol-fixed soft bodies of single dextral and sinistral individuals of *T.vestita*, which were available for the first time for interchiral comparison of genital morphology in the present genus, differed from each other in the pattern of penial microsculpture. They might represent enantiomorphs that have recently diverged in allopatry instead of enantiomorphism within a population or species. However, their shell and genital differences were not discrete enough to divide them taxonomically into two morphologically distinct species. Our results demonstrate the importance of evaluating individual variation relative to differences between incipient species in penial morphology, especially between conchologically indistinguishable enantiomorphs in the flat groups. We revise the taxonomy of the genus *Trichocathaica* including the above-mentioned new species, and *Trichocathaicaputeolata* Páll-Gergely, **sp. n.**

## Introduction

Left-right reversal of development seldom evolves in the Bilateria (Utsuno et al. 2011). Against this homochirality (directional asymmetry) rule of evolution, reversals of bilateral primary asymmetry, which visceral asymmetry represents, as well as secondary asymmetry such as coiling direction have recurrently evolved in gastropods (Okumura et al. 2008). Within gastropods, the evolution of left-right reversed species (chiral speciation) has been accelerated in terrestrial hermaphoroditic groups of the Stylommatophora (Gittenberger et al. 2012). At least in pulmonates, the left-right polarity of spiral cleavage, on which the direction of whole-body asymmetry depends, is determined by a maternal effect of a single nuclear gene. Thus, the chiral phenotype, dextral (clockwise-coiled) or sinistral (counter clockwise-coiled), exhibits maternal inheritance (Okumura et al. 2008; Asami et al. 2008; Utsuno and Asami 2010; Utsuno et al. 2010; Davison et al. 2016).

In stylommatophorans, their bimodal shell shapes are associated with the dichotomous mating modes. Groups with the flat/globular shaped shell copulate simultaneously reciprocally, whereas those with the tall shape copulate non-reciprocally, although exceptional cases are present (Asami 1993; Davison and Mordan 2007). In both of the modes, copulation between the reversed mutant and wild type (interchiral copulation) is less successful than intrachiral copulation with the same morph, which results in positive frequency-dependent selection (Johnson 1982). Interchiral copulation is less difficult in the non-reciprocal mode than in the simultaneous reciprocal mode (Gittenberger 1988; Asami et al. 1998). Thus, the mutant allele for reversal could persist longer under relaxed frequency-dependent selection in populations of tall species, which could then have a larger chance for fixation for the reversal allele, than in those of flat species. This prediction has been verified with observations of more frequent evolutions of reversed species in tall groups than in flat groups (Gittenberger 1988; Asami et al. 1998). Frequency-dependent selection against the less common chiral morph is therefore playing a key role for suppression of chiral speciation at least in stylommatophorans.

However, the frequency-dependent selection also plays a reverse role for chiral speciation, particularly in flat groups, which are subject to more stringent selection than the tall groups. Positive frequency-dependent selection works for the more frequent morph and against the less frequent opposite morph. This means that a population in spatial isolation could promptly be fixed for reversal once the reversed morph exceeds 50% in phenotypic frequency, for example through random genetic drift (Ueshima and Asami 2003) or survival advantage (Hoso et al. 2007, 2010). The homozygote of the recessive reversal mutant allele initially appears from mating between the heterozygotes. Because of the maternal inheritance, this homozygote develops the dominant phenotype. For example, in cases where the dextral wild-type is dominant to the sinistral mutant phenotype, crosses between the heterozygotes first generate the homozygotes of the sinistral allele, which develop into the dextral. These dextrals, having no mating difficulty with the dextral wild-type, generates only sinistral offspring. Thus, maternal inheritance could contribute to increase of the frequency of the reversed phenoype by drift. In the case of flat groups, population fixation for reversal in allopatry could establish premating reproductive (sexual) isolation from the other non-reversed populations, resulting in single-gene speciation (Gittenberger 1988; Orr 1991; van Batenburg and Gittenberger 1996; Ueshima and Asami 2003; Davison et al. 2005).

This stringency of frequency-dependent selection predicts that populations of flat groups are stably monomorphic for the direction of left-right asymmetry and that stably coexisting dextrals and sinistrals of flat snails are sexually isolated from each other. For example, a flat dextral camaenid ground-snail *Euhadraaomoriensis* evolved by reversal from the sinistral clade of *E.quesita*. Their shell surface morphologies have diverged since their speciation (Ueshima and Asami 2003). In accordance with the prediction, no evidence has validated chiral dimorphism (enantiomophism) that stably persists in a population of flat snails (Asami 1993; Asami et al. 1998).

However, as its name indicates, Möllendorff (1899) described a flat ground-snail *Trichocathaicaamphidroma* to have clockwise- or counterclockwise-coiled shell. The other congeners, *T.foliosquama* Wu, 2001, *T.goepeliana* Yen, 1938, *T.lyonsae* (Gude, 1919) *T.lyonsaecomosa* (Pilsbry, 1934) and *T.rugosobasis* (Pilsbry, 1934), are all described to be sinistral. In the Camaenidae, to which *Trichocathaica* belongs, many species perform simultaneous reciprocal mating, and no example of non-reciprocally mating has been found. Thus, most probably *Trichocathaica* snails also copulate simultaneously reciprocally. The taxonomical description of enantiomorphs as one species in this group (Möllendorff 1899) therefore poses the question, whether any morphological sign of divergence is detectable between them. Thus, the present study examined whether dextral and sinistral specimens of *T.amphidroma* exhibit differences in shell and/or genital traits of morphology.

Here we show the presence of enantiomorphs that are not distinguishable in shell surface morphology in each of three conchological species of *Trichocathaica*. We also taxonomically revise the *Trichocathaica* and describe two new species.

## Materials and methods

**Figure 1.**
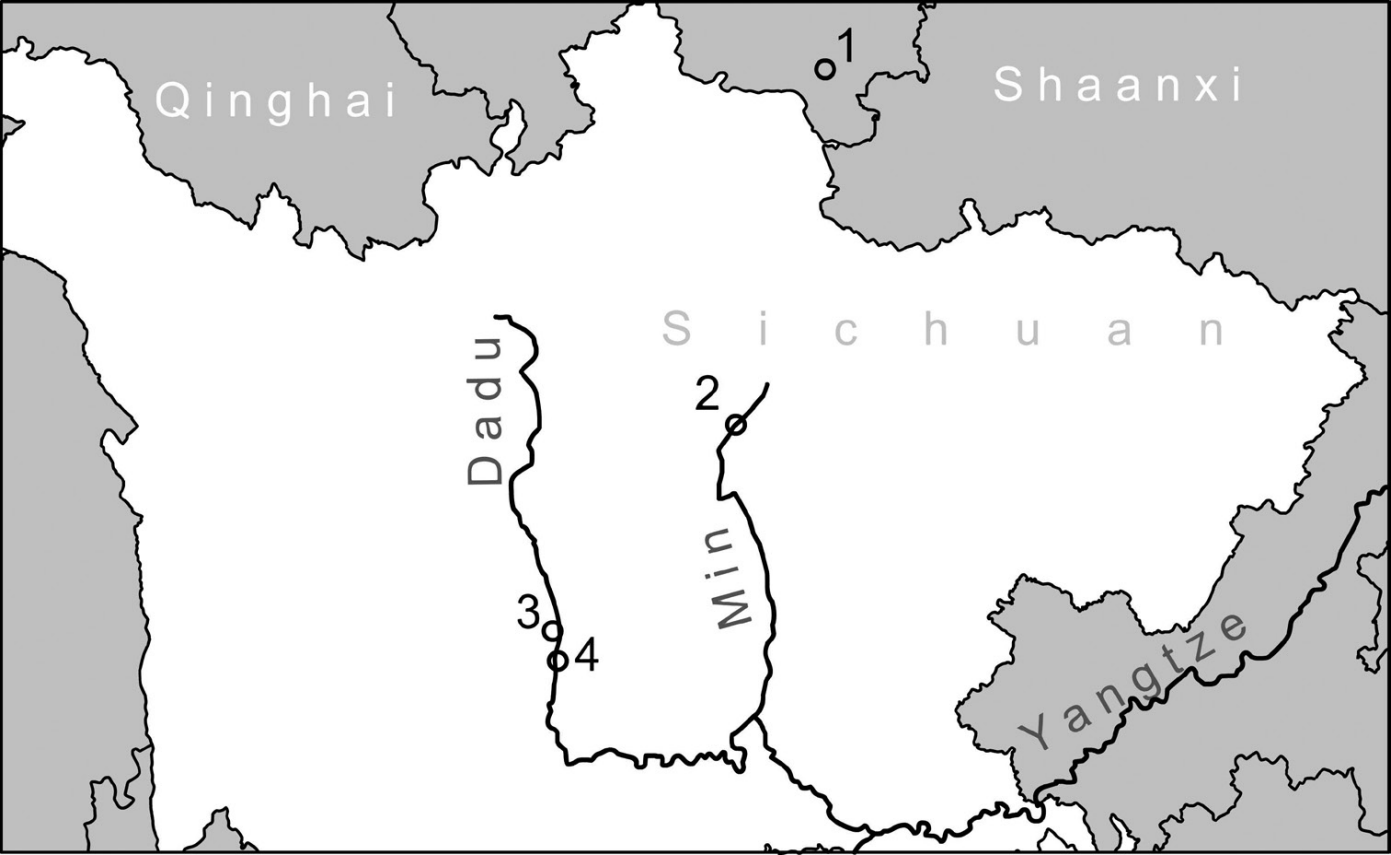
Map showing the exactly identifiable localities of *Trichocathaica* species **1** type locality of *Trichocathaicafoliosquama* Wu, 2001 **2** Type locality of *T.lyonsaecomosa* (Pilsbry, 1934) **3** Localities of *T.vestita* (2015/64 and 2015/65) **4** Localities of *T.amphidroma* (Möllendorff, 1899) (2015/67 and Wu 2001).

We examined shell and/or genital morphologies of specimens of the genus *Trichocathaica* available from the public and private collections listed below. Table 1 presents locality names cited verbatim from the specimen labels in the systematic part. Figure 1 shows the localities and rivers that are identifiable exactly on the map. A total of 155 shell specimens, two of which were deposited with ethanol-fixed soft-bodies, were available for our examination in eight species, including the two new species. One of the two with the soft-bodies was dextral and the other sinistral from one of the collecting localities, Wasihekou. We dissected these bodies for comparison of their genital morphology. We counted the whorls of each adult shell according to Kerney and Cameron (1979).

**Table 1.** Correspondence between locality names spelled on museum labels/literature and those in the present time.

Original	Present
Fulin Ton	Unknown
Ja sz’kou	Wasigou
Liu-ting	Luding
Lu Ho	Dadu River
Lu Tin Chouw	Unknown
Maochow	Unknown
Ta Tu Ho	Dadu River
Tapa	Unknown
Tapien	Unknown
Tung	Dadu River
Wa-sae-Kou	Wasigou
Wa-sy-kou	Wasigou

### Abbreviations

**ANSP** Academy of Natural Sciences (Philadelphia, USA)

**D** shell diameter

**H** shell height

**HA** Collection András Hunyadi (Budapest, Hungary)

**NHM** The Natural History Museum (London, UK)

**NHMUK** When citing NHM registered specimens

**PGB** Collection Barna Páll-Gergely (Mosonmagyaróvár, Hungary)

**SMF** Senckenberg Forschungsinstitut und Naturmuseum (Frankfurt am Main, Germany)

## Results

We found that all 72 specimens labelled as *Trichocathaicaamphidroma* were collected from the areas of Luding and Wasigou in west Sichuan, China. Five of the nine lots included both clockwise-coiled (dextral) and counter clockwise-coiled (sinistral) specimens. In total, 39 were dextral and 33 were sinistral. However, our further examination of shell morphology revealed that they include three distinct morphotypes that differ in fine sculpture of the shell surface, especially in the size of the periostracal scales. The dextral and sinistral soft-bodies exhibited no distinct differences in the gross anatomy of the genital system. However, the patterns of microsculpture in the internal surface of the penial tube were slightly different between them.

There were 28, 5 and 29 shells corresponding to the three morphotypes. We found no example of intermediate morphology in shell traits between those morphotypes. Each of the three morphotypes was present in mixture with one of the other morphotypes in four different lots of SMF collection. This suggests that those pairs of morphotypes probably exhibit their discrete shell differences in sympatry as well. On the other hand, the differences of penial sculpture were detected between only single specimens available in each of enantiomorphs. Their shells did not differ in traits that divide the three morphotypes. For these reasons, we recognize the three morphotypes as distinct species, *T.amphidroma*, *T.macrosquamata* sp. n. and *T.vestita* (Pilsbry, 1934). We classified the dextral and sinistral individuals, the penial sculpture patterns of which were examined, in *T.vestita* and describe their penial difference. Deviation of the morph frequency from 0.5 was not statistically significant in 28 specimens of *T.amphidroma* (P = 0.06, χ^2^ = 3.6) (Table 2). In contrast, dextrals were more frequent than sinistrals in 39 specimens of *T.vestita* (P = 0.002, χ^2^ = 9.3). Between these species, sinistrals were more frequent in *T.amphidroma* (P = 0.001, Fisher’s exact test).

**Table 2.** Numbers of enantiomophic specimens found in three morphological species.

Locality	Collecting year	* T. amphidroma *	*T.macrosquamata* sp. n.	* T. vestita *
“Liu-Ting am Tung” (Luding at Dadu River)	1884–1886	2 sinistrals	–	2 dextrals
“Thal des Tung” (Valley of Dadu River)	1884–1886	3 sinistrals	–	
Luding (2015/67)	2015	6 sinistrals	–	
“Ta Tu Ho” (Dadu River)	1930	8 sinistrals 9 dextrals	–	2 dextrals
“Wa-sae-Kou” (Wasigou)	probably 1884–1886	–	1 sinistral 1 dextral	3 dextrals
“Wa-sy-Kou am Tung” (Wasigou at Dadu River)	1884–1886	–	2 sinistrals	–
“W-Sytshuan” (West Sichuan)	1884–1886	–	1 sinistral	1 dextral
“Wasihekou” (Wasigou, 2015/64)	2015	–	–	19 dextrals
“Wasihekou” (Wasigou, 2015/65)	2015	–	–	10 sinistrals 2 dextrals
Total		19 sinistrals 9 dextrals	4 sinistrals 1 dextral	10 sinistrals 29 dextrals

### Systematic part

#### Family Camaenidae

Taxon classificationAnimaliaStylommatophoraCamaenidae

Pilsbry, 1895

##### Remarks.

Camaenidae and Bradybaenidae are traditionally distinguished on the basis of the absence of the dart sac and mucous glands in the former and the presence of these structures in the latter. The molecular phylogeny of Wade et al. (2007) showed that the dart sac was lost multiple times during the evolution of the Camaenidae. Camaenidae and Bradybaenidae form a single clade, and neither of them is monophyletic. Therefore Gittenberger et al. (2012) formally treated the Bradybaenidae as a junior synonym of Camaenidae. Bouchet et al. (2017) retained the subfamily Bradybaeninae Pilsbry, 1934 under Camaenidae. This system is followed here.

#### Genus Trichocathaica

Taxon classificationAnimaliaStylommatophoraCamaenidae

Gude, 1919

Cathaica (Trichocathaica) Gude, 1919: 119.

##### Type species.

Cathaica (Trichocathaica) lyonsae Gude, 1919, by original designation.

##### Distribution.

All *Trichocathaica* are known from the eastern edge of the Tibetan Plateau in the Chinese Sichuan and Gansu provinces (valleys of the Dadu and Min rivers).

##### Remarks.

Wu (2015) mentioned *Trichocathaicamaoensis*, but this name has not been made available.

#### Enantiomorphic species

##### Trichocathaica amphidroma

Taxon classificationAnimaliaStylommatophoraCamaenidae

(Möllendorff, 1899)

Figure 2

**Figure 2.**
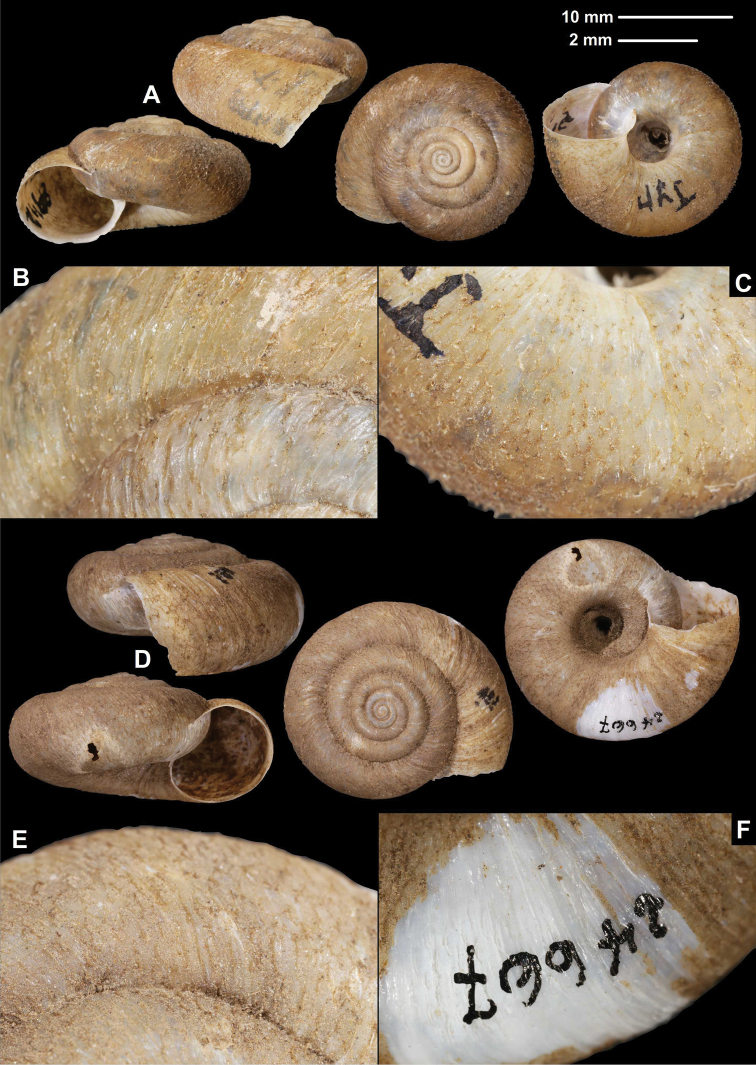
Shells (**A, D**), dorsal (**B, E**), and ventral (**C, F**) sculpture of *Trichocathaicaamphidroma* (Möllendorff, 1899) **A–C**SMF 8942 (lectotype, D = 20.8 mm) **D–F**SMF 24667 (paralectotype, D = 23.6 mm). Scale bars: Upper scale (**A, D**) lower scale (**B, C, E, F**). All photos: B. Páll-Gergely.

**Figure 3.**
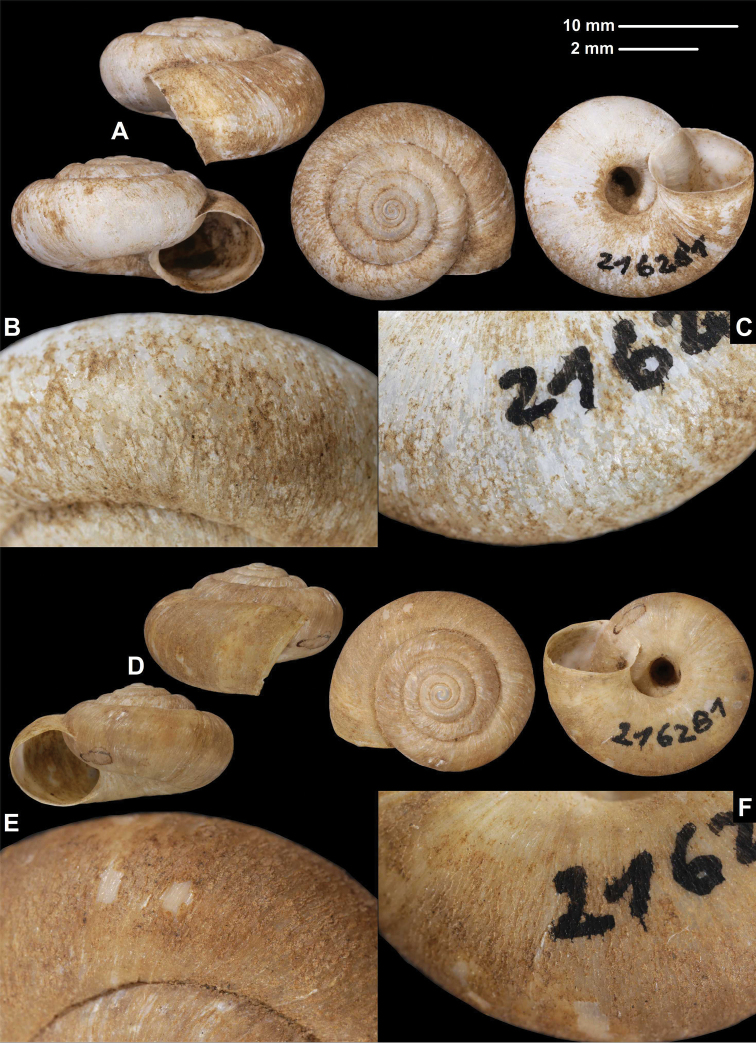
Shells (**A, D**), dorsal (**B, E**), and ventral (**C, F**) sculpture of *Trichocathaicamacrosquamata* Páll-Gergely sp. n. **A–C**SMF 349504 (paratype, D = 21.8 mm) **D–F**SMF 216281 (holotype, D = 18.8 mm). Scale bars: Upper scale (**A, D**), lower scale (**B, C, E, F**). All photos: B. Páll-Gergely.

Euhadra amphidroma Möllendorff, 1899: 83, plate 4, figs 2, 2a, 3.
Trichocathaica amphidroma – Yen 1939: 150, plate 15, fig. 44.
Trichocathaica amphidroma – Yen 1939: 292, figs 1–4.

###### Material examined.

W-Sytschuan, Liu-Ting am Tung, coll. Möllendorff ex coll. Potanin, SMF 8942/1 lectotype (sinistral shell, D = 20.8 mm, H = 11.6 mm, Fig. 2A–C); same data, SMF 8938/1 paralectotype (sinistral shell); W-Sytschuan, Thal des Tung, coll. Möllendorff ex coll. Potanin, SMF 8937a/3 sinistral, juvenile paralectotype shells; China (Szytschuan): W-Ufer d. Lu Ho (Ta Tu Ho) zw. Tapien und Ja sz’kou, ex Krejci-Graf, 05.08.1930, SMF 24667/8 sinistral + 9 dextral shells (1 of the dextral shells is photographed: Fig. 2D–F); **2015/67** Sichuan, Ganzi Zhou, Luding Xian, Luding, rocks above bus station, approximate GPS coordinates: 29°54.451'N, 102°13.923'E, leg. Hunyadi, A., 15.06.2015, HA/6 sinistral shells.

###### Diagnosis.

Shell sinistral or dextral, body whorl rounded, teleoconch roughly wrinkled with large, triangular periostracal folds, fold scars represented as long curved lines.

###### Description.

Shell sinistral or dextral, spire slightly elevated, body whorl rounded, protoconch consists of 1.25–1.5 whorls, finely, irregularly wrinkled; entire shell with 5.5–5.75 whorls; teleoconch roughly, irregularly wrinkled, with large, triangular periostracal folds having long, curved (C-shaped) base; in specimens/shell parts without periostracum the base of folds visible as prominent curved lines on the surface; aperture subcircular, peristome slightly expanded, thin, sharp; inner thickening parallel to peristome weak.

###### Measurements (in mm).

D = 20.5–25.9, H = 10.3–13.1 (*n* = 6).

###### Differential diagnosis.

The fine sculpture of the teleoconch surface, namely the rough wrinkles and large triangular periostracal folds with the long base, distinguish this species form the other congeners.

###### Distribution.

This species is known from the valley of the Dadu River near Luding. We were not able to locate Tapien on a map. Wu (2001) also reported this species from Luding County (29.9°N, 102.2°E).

##### Trichocathaica macrosquamata

Taxon classificationAnimaliaStylommatophoraCamaenidae

Páll-Gergely sp. n.

http://zoobank.org/C0AE35B8-C9AC-486D-AC75-FA21FF03C1BD

Figure 3

###### Material examined.

Setschuan, Wa-sae-Kou, coll. Jaeckel ex coll. Schäfer, SMF 216281, holotype (1 sinistral shell, D = 18.8 m, H = 10.6 mm, Fig. 3D–F); same data, SMF 349504, paratype (1 dextral shell, Fig. 3A–C); China, W-Sytschuan: Wa-sy-Kou am Tung, coll. O. Möllendorff ex coll. Potanin, SMF 8941, paratypes (2 sinistral shells, paralectotypes of *amphidroma*, 1 of them is corroded without hair scars); W-Sy-tschuan, SMF 9171, paratype (1 sinistral shell, paralectotype of *amphidroma*).

###### Diagnosis.

Shell sinistral or dextral, body whorl rounded with a very slight indication of a keel, teleoconch finely wrinkled with medium-sized scale-like periostracal folds; fold scars represented as medium-sized curved lines.

###### Description.

Shell sinistral or dextral, spire slightly elevated, body whorl rounded with a very slight indication of a keel; protoconch consists of 1.25–1.5 whorls, finely, irregularly wrinkled; entire shell with 5.5–6 whorls; teleoconch finely, irregularly wrinkled, with medium-sized, low, dense periostracal folds having curved (C-shaped) base; scales visible to the naked eye; in specimens/shell parts without periostracum the bases of folds visible as curved lines; aperture subcircular, peristome slightly expanded, thin, sharp; inner, white thickening parallel to the peristome prominent, situated in some distance from peristome edge.

###### Measurements (in mm).

D = 18.8–23.7, H = 10.6–13 (*n* =4).

###### Differential diagnosis.

*Trichocathaicamacrosquamata* sp. n. differs from *T.vestita* by exhibiting larger periostracal folds (scales) over the entire shell surface.

**Etymology.** This species is named after its scales on the shell surface, which are larger than those of *T.vestita*.

###### Distribution.

This species is known from the valley of the Dadu River at Wasigou.

##### Trichocathaica vestita

Taxon classificationAnimaliaStylommatophoraCamaenidae

(Pilsbry, 1934) comb. n.

**Figure 4.**
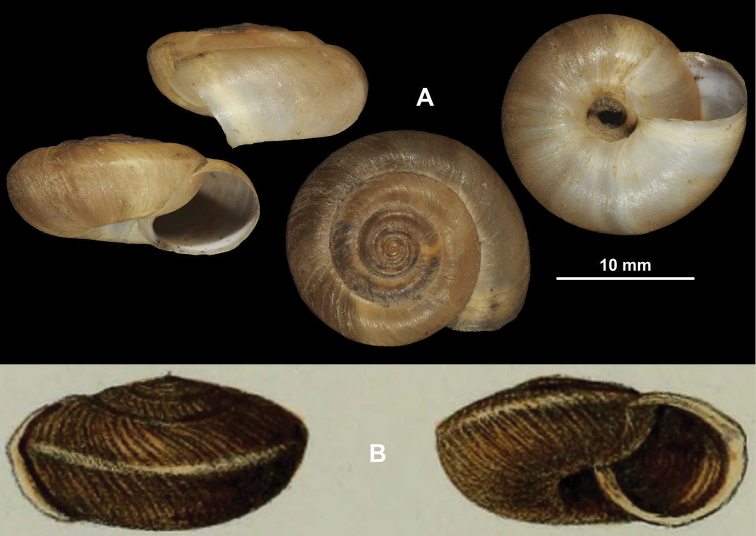
Shells of *Trichocathaicavestita* (Pilsbry, 1934) (holotype, ANSP 159708) (**A**) and Helix (Camaena) constantinae Adams, 1870 (**B**) (illustration from the original description). Scale bars: Scale refers only to **A**.

**Figure 5.**
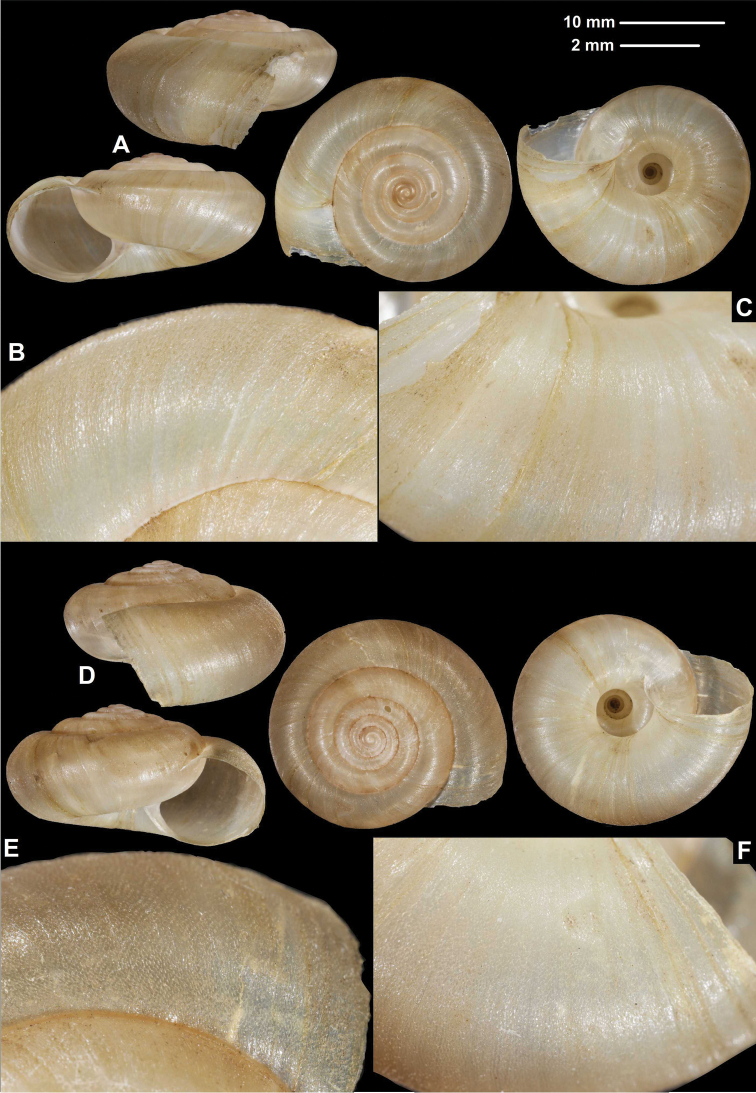
Shells (**A, D**), dorsal (**B, E**), and ventral (**C, F**) sculpture of *Trichocathaicavestita***A–C** HNHM 103471 (D = 22.7 mm) **D–F** HNHM 103470 (D = 22.8 mm). Upper scale (**A, D**), lower scale (**B, C, E, F**). All photos: B. Páll-Gergely.

**Figure 6.**
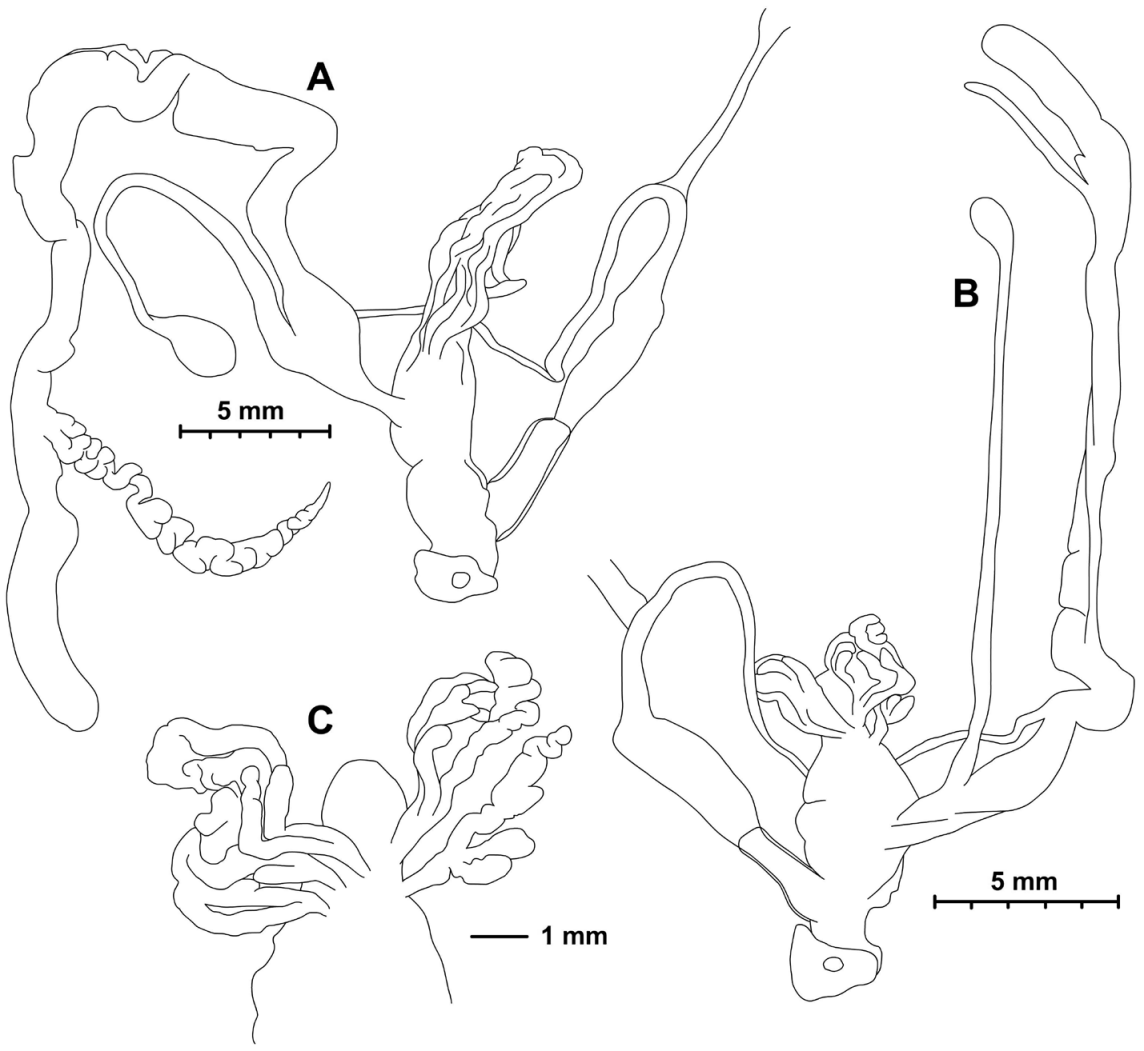
Genital anatomy of *Trichocathaicavestita***A** Dextral specimen (HNHM 103470) **B, C** Sinistral specimen (HNHM 103471).

**Figure 7.**
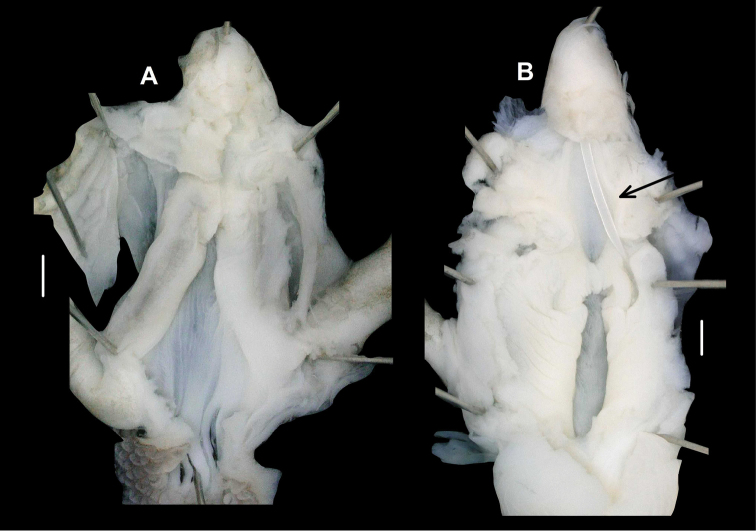
Opened dart sac of sculpture of *Trichocathaicavestita***A** Dextral specimen (HNHM 103470) **B, C** Sinistral specimen (HNHM 103471). Arrow shows dart. Scale bars: 1 mm.

**Figure 8.**
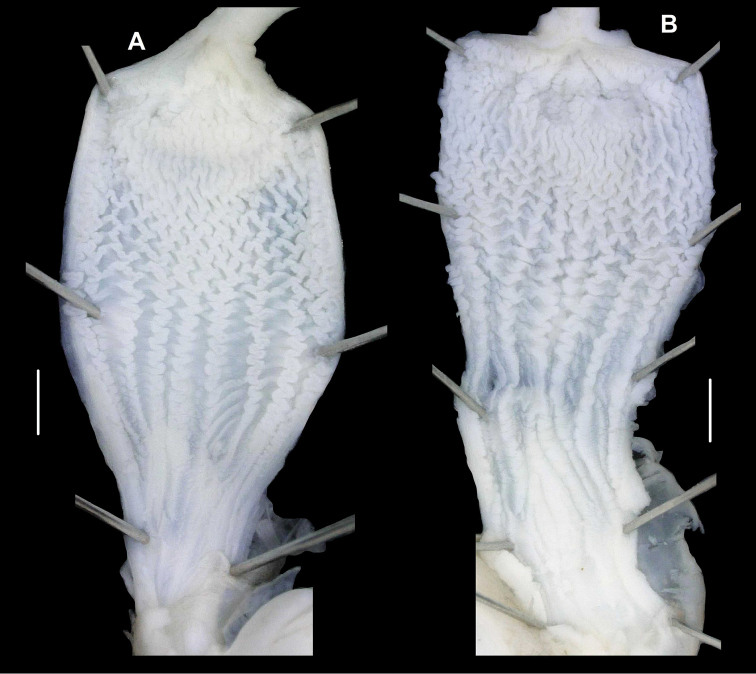
Penial sculpture of *Trichocathaicavestita***A** Dextral specimen (HNHM 103470) **B, C** Sinistral specimen (HNHM 103471). Scale bars: 1 mm.

Figures 4A , 5 , 6 , 7 , 8

Cathaica constantinae vestita Pilsbry, 1934: 15, plate 3, figs 5–7.

###### Types examined.

Between Wenchwan and Weichow, June, 1931, ANSP 159708 (photos of the holotype were examined).

###### Additional material examined.

**2015/64** Sichuan, Ganzi Zhou, Kangding Xian, Wasihekou, southern side of the river, along the highway, 1420 m a.s.l., 30°04.564'N 102°09.865'E, leg. A. Hunyadi, 14.06.2015, HNHM 103470 (dextral shell, Fig. 5D–F + ethanol-preserved body: Figs 6A, 7A, 8A), HA/15 dextral shells, PGB/3 dextral shells; **2015/65** China, Sichuan, Ganzi Zhou, Kangding Xian, Wasihekou 200 m towards Guzan Zhen, around the stupa, 1420 m a.s.l., 30°04.565'N, 102°10.085'E, leg. Hunyadi, A. & Szekeres, M., 14.06.2015, HNHM 103471 (1 sinistral shell, Fig. 5A–C + ethanol-preserved body: Figs 6B–C, 7B, 8B), HA/6 sinistral shells + 2 dextral shells, PGB/3 sinistral shells; W-Sytschuan, Wa-sy-Kou am Tung, coll. Möllendorff ex coll. Potanin, SMF 8940 (1 dextral shell, paralectotype of *amphidroma*); China, W-Sytschuan: Wa-sy-Kou am Tung, coll. O. Möllendorff ex coll. Potanin, SMF 349506 (1 dextral shell, paralectotype of *amphidroma*, ex SMF 8941); Sy-tschuan, rechter Ufer des Flusses Tun bei dem Torfe (?) Wa-sy-ku, coll. O. Möllendorff ex coll. Potanin 3898a, 1903, SMF 95002 (1 dextral shell, paralectotype of *amphidroma*); W-Sy-tschuan, coll. Möllendorff, SMF 349505 (1 dextral shell, paralectotype of *amphidroma*, ex SMF 9171); W-Sytschuan, Liu-Ting am Tung, coll. Möllendorff ex coll. Potanin, SMF 349503 (2 dextral shells, paralectotypes of *amphidroma*, ex SMF 8938); W-Ufer d. Lu Ho (Ta Tu Ho) zw. Tapien und Ja sz’kou, ex Krejci-Graf, 05.08.1930, SMF 349507 (2 dextral shells, ex SMF 24667).

###### Diagnosis.

Shell sinistral or dextral, body whorl rounded to keeled, teleoconch finely wrinkled with small scale-like periostracal folds; fold scars (if visible) represented as short curved lines.

###### Description.

Shell sinistral or dextral, spire slightly elevated; body whorl rounded (with a very slight indication of a keel) to keeled, protoconch consists of 1.25–1.5 whorls, finely, irregularly wrinkled; entire shell with 5.25–5.75 whorls; teleoconch finely, irregularly wrinkled, with small, low, dense periostracal folds having curved (C-shaped) base; scales not visible to the naked eye; in specimens/shell parts without periostracum the bases of folds or sometimes not visible as small curved lines; aperture subcircular, peristome slightly expanded, thin, sharp; inner, white thickening parallel to the peristome prominent, situated in some distance from peristome edge.

###### Measurements (in mm).

D = 21.2–23.6, H = 11.1–13.6 (*n* = 12).

###### Anatomy

(Figs 6, 7). Genital morphology of two specimens (HNHM 103470, dextral specimen and HNHM 103471, sinistral specimen) showed that the left ommatophoral retractor crosses between penis and vagina in the sinistral specimen, and that the right retractor in the dextral specimen. Atrium short, penis with a slimmer, shorter distal, and a thicker, longer proximal portion, distal portion covered by a weak penial sheath; epiphallus much more slender than penis, approximately as long as penis; retractor muscle shorter in sinistral and longer in dextral specimen, inserts on epiphallus, close to its meeting point with penis; proximal part of penis internally with reticulated zigzag sculpture caused by the perpendicular projections of longitudinal folds (Fig. 8); dart sac well developed, with thickened, larger basal part and smaller head part; dart was only found in the dextral specimen (Fig. 7); long glandulae insert on 4–6 points on the “neck” of the dart sac (at the meeting point of the body and head of the dart sac) (Fig. 6C); vagina short in dextral and longer in sinistral specimen, stalk of bursa copulatrix long, relatively slender, bursa ovoid, diverticulum absent; spermoviduct slender, no embryos found; albumen gland crescent shaped, talon relatively large.

We found no discrete differences between the dextral and sinistral individuals in gross anatomy of the genital system or in the internal structure of the dart sac (Figs 6, 7). The internal surface of the penial tube exhibited complex patterns of microsculpture typical to camaenid snails (Fig. 8). In this structure, several slight differences were noticeable between these specimens of enantiomorphs. Around the area leading to the epiphallus (upper edge in Fig. 8), thin longitudinal pilasters are more tightly gathered with narrower furrows in the dextral than in the sinistral. In both of them, toward the middle of the penial tube, these pilasters become thick and sparse with wider furrows and form a reticulate pattern. Longitudinally (in the direction towards the genital orifice, the bottom in Fig. 8) under the reticulated range, zigzag crenulated pilasters are present in parallel. In these portions, structural change from the reticulate pattern to the parallel pilasters is more distinct in the dextral than in the sinistral. Smooth-bottomed furrows separate the zigzag pilasters in the dextral, whereas those furrows are not obviously present between the irregularly zigzag-shaped pilasters in the sinistral. This structure of the dextral is present in a longitudinally wider range than the sinistral. In the sinistral, instead, the irregular zigzag structure becomes weak or disappears from the longitudinal pilasters, which become thick and pronounced near the genital orifice. This longitudinal structural change is not present in the dextral. The dextral instead exhibits a different pattern such that the longitudinally parallel zigzag pilasters are continuously present and merge with one another without forming major pilasters.

###### Differential diagnosis.

*Trichocathaicavestita* differs from *T.macrosquamata* sp. n. by having the smaller periostracal folds (scales) on the entire shell surface.

###### Distribution.

This species is known from the valley of the Dadu River at Luding and Wasigou.

###### Remarks.

This species was described as a subspecies of Helix (Camaena) constantinae Adams, 1870. We had no possibility to examine that species; however, it has remarkably different shell traits, such as the strongly sculptured shell surface and a white band (see Fig. 4B). That species is probably not a *Trichocathaica*, but something entirely different, as Pilsbry (1934) already suspected. Moreover, *Trichocathaica* seems to inhabit only the mountains in Sichuan and southern Gansu, and Helix (Camaena) constantinae was collected in the “Ichang gorge” on the Yangtze River in Hubei Province (ca 30°55'N, 110°50'E, Adams 1870). Therefore, we handle *Trichocathaicavestita* (Pilsbry, 1934) as a species of its own right.

Pilsbry (1934) already noted in the original description that the species show an extreme variability in terms of the development of the keel. Our data also indicates that the keel morphology is variable within and between populations. In the sample of 2015/64, 15 shells had nearly rounded body whorls, two were keeled, and two were intermediate between those rounded and keeled forms. Although every shell in the sample of 2015/65 had a keel, two of them were similar to the intermediate form of the 2015/64 sample.

#### Sinistral species

##### Trichocathaica foliosquama

Taxon classificationAnimaliaStylommatophoraCamaenidae

Wu, 2001

Trichocathaica foliosquama Wu, 2001: 294, figs 5–13.

###### Type locality.

Lijiexiang, Zhouqu County (33.8N, 104.3E), Gansu Province.

###### Description

(based on the original description and photos). Shell sinistral, spire slightly elevated, body whorl rounded with a very slight indication of a blunt keel; protoconch consists of 1.55–1.75 whorls, finely granulose; entire shell with 5–5.38 whorls; teleoconch roughly, irregularly wrinkled, with large scales, each scale with 2 or 3 lamellae around 1 central ridge; aperture subcircular, peristome slightly expanded, thin, sharp; inner thickening parallel to peristome situated in some distance from peristome edge.

###### Measurements

(in mm, based on the original description). D = 17.03–18.48, H = 9.33–10.35.

###### Distribution.

This species is known from the type locality only.

###### Remarks.

We did not examine specimens of this species. It can be distinguished from the other species by the narrow umbilicus and the morphology of periostracal folds, described as “shell surface scaly, and each scale with 2–3 lamellae around 1 central ridge” (Wu 2001).

##### Trichocathaica goepeliana

Taxon classificationAnimaliaStylommatophoraCamaenidae

Yen, 1938

Figure 9

Trichocathaica goepeliana – Yen 1939: 150, plate 15, fig. 45.

###### Material examined.

W-Sy-tschuan: Tapa, coll. Möllendorff ex Potanin 444, SMF 8939/2 (syntypes).

###### Diagnosis.

Shell sinistral, small, body whorl keeled at the middle of body whorl, teleoconch finely wrinkled with small scales.

###### Description.

Shell small, sinistral, spire elevated, dorsal surface domed/conical; body whorl bluntly keeled, keel situated at about mid part of body whorl; shallow subsutural furrow present on body whorl above keel; protoconch consists of 1.5 whorls, finely pitted and wrinkled; entire shell with 5.5–5.75 whorls; teleoconch finely, irregularly wrinkled, with fine, curved scales (present near suture); aperture subcircular, peristome not developed in the two available specimens.

**Figure 9.**
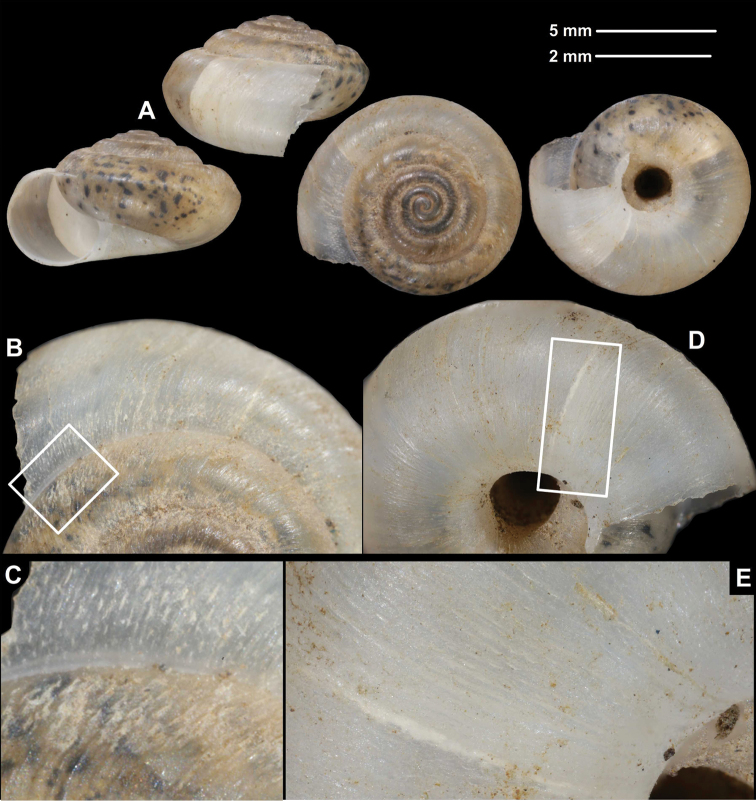
Lectotype of *Trichocathaicagoepeliana* Yen, 1938 (SMF 8939, syntype, D = 9.6 mm). **A** Entire shell **B, C** Dorsal sculpture **D, E** Ventral sculpture **C, E** show white framed area of **B** and **D** respectively. Scale bars: Upper scale (**A**), lower scale (**B, D**) **C, E** not to scale. All photos: B. Páll-Gergely.

###### Measurements (in mm).

D = 9.5–9.6, H = 5.7–6.1 (*n* = 2).

###### Differential diagnosis.

This species is smaller than the other congeners, and its keel is situated in the middle of the body whorl.

###### Distribution.

This species is known from the type locality only. We were not able to determine the locality Tapa on the map among more than 20 similarly called localities.

###### Remarks.

This species differs from the other congeners by its smaller shell; both the available shells are smaller than 10 mm in diameter, whereas the others have shells larger than 15 mm. Though the small scales on the shell surface suggest that this species belongs to the genus *Trichocathaica*, due to its small shell size, its generic placement needs to be verified by examination of genital morphology.

##### Trichocathaica lyonsae

Taxon classificationAnimaliaStylommatophoraCamaenidae

(Gude, 1919)

###### Remarks.

This species can be distinguished from the other congeners by the long and slender hairs, which are mostly present on the side of the body whorl and the upper edge of the preceding whorls (i.e. inside the suture). We received photos of the types of the two subspecies, and therefore, their fine sculpture could not be examined.

###### Distribution.

This species is only known from the valley of the Min River.

##### Trichocathaica lyonsae lyonsae

Taxon classificationAnimaliaStylommatophoraCamaenidae

(Gude, 1919)

Figure 10A–C

Cathaica (Trichocathaica) lyonsae Gude, 1919: 119 + unnumbered figure.
Trichocathaica lyonsae – Yen 1939: 150, plate 15, fig. 43.

###### Types examined.

Min Valley, Setchuen, coll. Gude, NHMUK 1922.8.29.86 (syntype).

###### Type locality.

“Min Valley, Setchuen”.

###### Diagnosis.

Shell sinistral, body whorl slightly shouldered, teleoconch roughly wrinkled with medium-sized scale-like periostracal folds; some folds are developed to long hairs.

###### Description.

Shell sinistral, spire slightly elevated, dorsal side rather low conical; body whorl slightly shouldered; protoconch consists of 1.5 whorls, finely, irregularly wrinkled; entire shell with 5.5 whorls; teleoconch roughly, irregularly wrinkled, with moderately large, triangular periostracal folds; some folds on the edge of body whorl and in the suture developed to long, cylindrical hairs; in shell parts without periostracum the base of folds visible as deep scars; aperture subcircular, slightly ovoid (depressed in dorsobasal direction); upper peristome edge strongly descending (mostly visible from lateral view); peristome slightly expanded, sharp; inner thickening parallel to the peristome strong, white.

**Figure 10.**
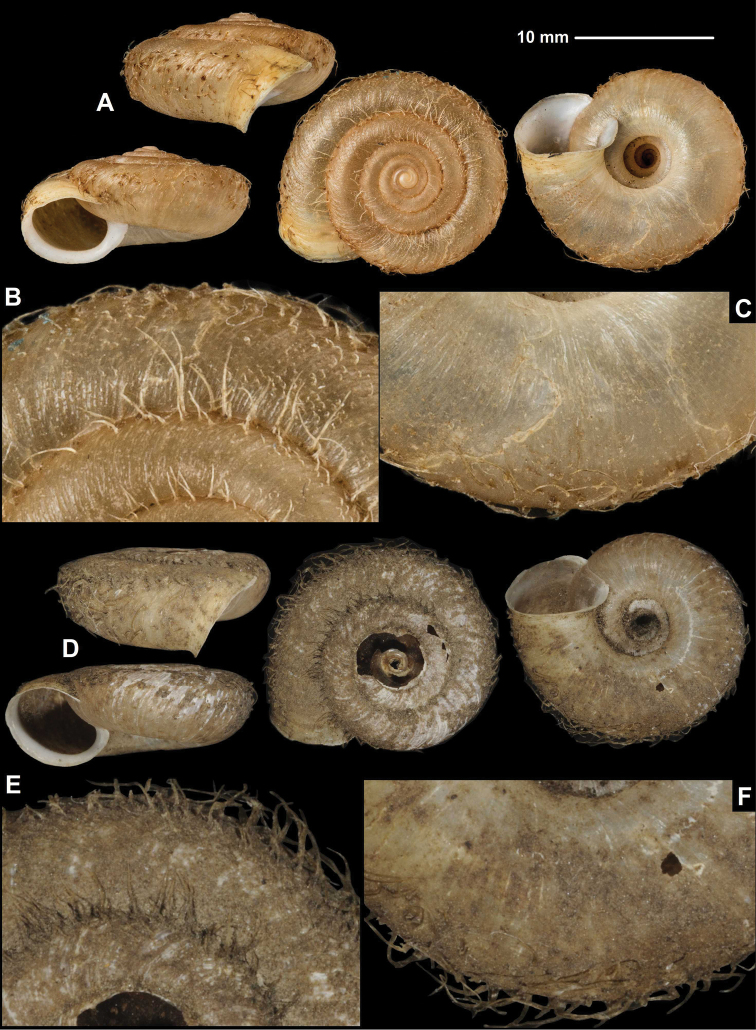
**A–C***Trichocathaicalyonsaelyonsae* (Gude, 1919), NHMUK 1922.8.29.86 (syntype, D = 17.3 mm) **D–F***Trichocathaicalyonsaecomosa* (Pilsbry, 1934), ANSP 159641 (holotype, D = 15.7 mm). Close-up images show the dorsal (**B, E**), and the ventral (**C, F**) sculpture. Scale refers to **A, D** other figures not to scale. Photos: K. Seizova, ANSP Malacology Department (**D–F**) and H. Taylor (**A–C**).

###### Measurements (in mm).

D = 17.3, H = 8.7 (photographed syntype).

###### Distribution.

This subspecies is known from the type locality only.

##### Trichocathaica lyonsae comosa

Taxon classificationAnimaliaStylommatophoraCamaenidae

(Pilsbry, 1934)

Figure 10D–E

Bradybaena (Trichocathaica) lyonsaecomosa Pilsbry, 1934: 86: 11–12, plate 4, figs 4, 5.

###### Types examined.

Wenchwan, Szechuan, China, on a dry hill slope, (W. China exp., Brooke Dolan, 24.04.1931), ANSP 159641 (holotype).

###### Type locality.

“Wenchwan, Szechuan, on a dry hill slope”; “between Kwanhsien and Yuchi”.

###### Measurements (in mm).

D = 15.7, H = 7.0 (*n* = 1, according to the original description).

###### Differential diagnosis.

*Trichocathaicalyonsaecomosa* differs from the nominotypical subspecies by the slightly smaller shell, the less descending aperture and the flat dorsal side.

##### Trichocathaica puteolata

Taxon classificationAnimaliaStylommatophoraCamaenidae

Páll-Gergely sp. n.

http://zoobank.org/C89554FE-A309-42C5-BDC5-328153FB7EF6

Figure 11A–C

Laeocathaica (Trichocathaica) lyonsae – Wenz 1960: 639, fig. 2235.

###### Material examined.

China, Sytschuan, “Hügel bei der Fähre aus Ta Tu Ho bei %” (one side of the label), “Fulin Ton boden, Maisfelden, 31.07.1930” (other side of the label), SMF 24666a/1 (holotype, orig. Handb. Pal. 2235); same data, SMF 349516/61 shells (most of them are juveniles); same data, SMF 349514/1 paratype shell (orig. Yen, 1939: plate 15, fig. 43); China, Sytschuan, Osthang des Lu-ho (=Ta Tu-Ho), s. Lu Tin Chouw (?), ex coll. K. Krejci-Graf, 04,08,1930 (1933), SMF 24665/15 (most of them are juveniles).

###### Diagnosis.

Shell large, sinistral, body whorl keeled above mid-part of body whorl, teleoconch roughly wrinkled with moderately large, slender periostracal folds, fold scars represented as deep pits.

###### Description.

Shell sinistral, spire very slightly elevated in most specimens (dorsal side nearly flattened), but in some shells dorsal surface domed; body whorl slightly keeled, keel situated above mid part of body whorl; protoconch consists of 1.25–1.75 whorls, finely, irregularly wrinkled; entire shell with 4.5–5.5 whorls; teleoconch roughly, irregularly wrinkled, with moderately large, slender triangular, sometimes cylindrical, hair-like periostracal folds having short, curved base; in specimens/shell parts without periostracum the base of folds visible as deep fold scars; aperture subcircular, peristome slightly expanded, thin, sharp; inner thickening parallel to the peristome weak.

**Figure 11.**
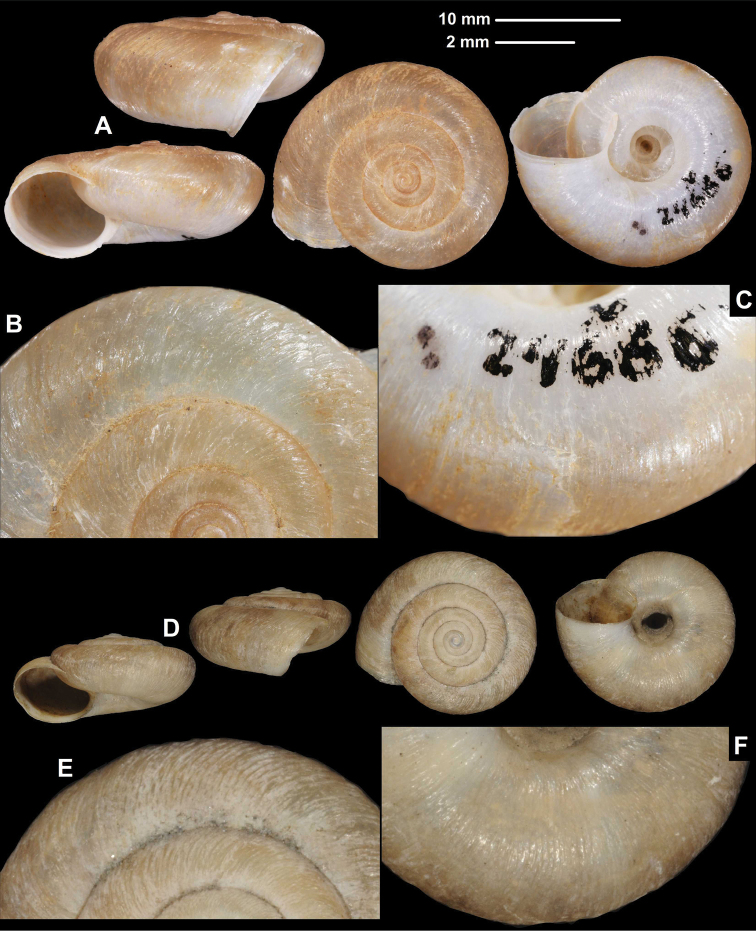
Shells (**A, D**), dorsal (**B, E**), and ventral (**C, F**) sculpture of *Trichocathaica* species **A–C***Trichocathaicarugosobasis* (Pilsbry 1934), ANSP 159636 (holotype, D = 15 mm) **D–F***Trichocathaicaputeolata* Páll-Gergely sp. n., holotype (SMF 24666, D = 19.5 mm). Scale bars: Upper scale (**A, D**); lower scale (**B, C, E, F**). Photos: B. Páll-Gergely (**D–F**) and K. Seizova, ANSP Malacology Department (**A–C**).

###### Measurements (in mm).

D = 15.7–22.8, H = 7.5–11.4 (*n* = 6).

###### Differential diagnosis.

The shell of *T.rugosobasis* is similar to that of *T.puteolata* sp. n. but smaller with the narrower umbilicus, less keeled body whorl, and stronger radial sculpture.

###### Etymology.

The Latin puteolata (= pitted) refers to the pitted surface of the shells.

###### Distribution.

This species is known only from the valley of the Dadu River.

###### Remarks.

All shells we examined in the SMF were labelled as *T.lyonsae*.

##### Trichocathaica rugosobasis

Taxon classificationAnimaliaStylommatophoraCamaenidae

(Pilsbry, 1934)

Figure 11D–F

Bradybaena (Trichocathaica) rugosobasis Pilsbry, 1934: 86: 12, plate 4, figs 6, 7.

###### Type examined.

Wenchwan to Maochow, Szechuan, China (W. China exp., Brooke Dolan, 24.04.1931), ANSP 159636 (holotype [photos examined] + 2 paratypes [not examined]).

###### Type locality.

“Between Wenchwan and Maochow, Szechuan, on rocks in the arid valley of the Min River, 3500–4100 ft. elevation”.

###### Description

**(based on photos of the holotype).** Shell sinistral, spire slightly elevated, body whorl rounded with a very slight indication of a blunt keel; protoconch consists of 1.75 whorls, probably finely, irregularly wrinkled; entire shell with 5.75 whorls; teleoconch very roughly, irregularly wrinkled, with large hair scars, periostracal elements not visible; aperture subcircular, peristome slightly expanded, thin, sharp; inner thickening parallel to peristome seemingly normally developed, situated in some distance from peristome edge.

###### Measurements (in mm).

D = 14.3–15.0, H = 7.0–7.2 (*n* = 2, according to the original description).

###### Remarks.

This species is characterized by its medium-sized shell with rough radial sculpture. The holotype is somewhat corroded, and thus, the periostracal folds could not be examined.

##### Trichocathaica

Taxon classificationAnimaliaStylommatophoraCamaenidae

sp.

Figure 12

###### Material examined.

W-Sytschuan, Thal des Tung, coll. Möllendorff ex coll. Potanin, SMF 8937/1 sinistral shell.

###### Measurements (in mm).

D = 20.2, H = 12.0 (*n* = 1).

###### Remarks.

The single shell specimen is not corroded and has no scales/hairs on its surface. Thus, this specimen might be of an undescribed species. However, we hesitate to describe this taxon until more specimens become available.

**Figure 12.**
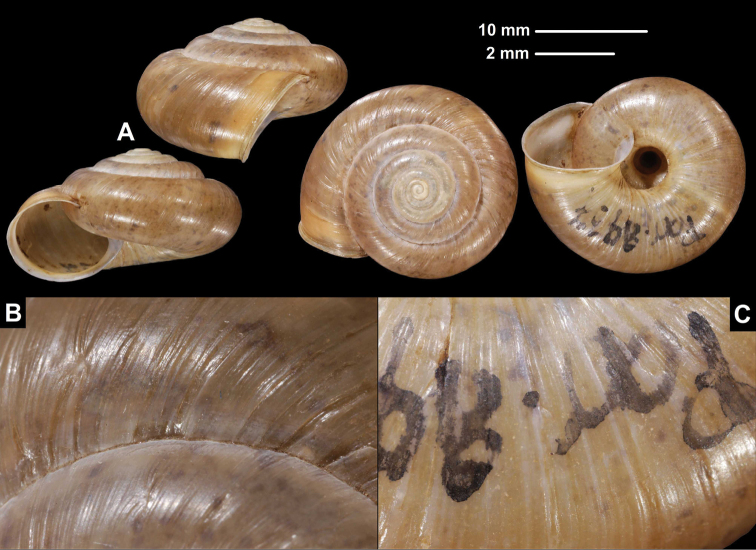
Shell (**A**), dorsal (**B**), and ventral (**C**) sculpture of *Trichocathaica* sp. (SMF 8937, D = 20.2 mm). Scale bars: Upper scale (**A**), lower scale (**B, C**). All photos by B. Páll-Gergely.

## Discussion

The present study discovered that both dextral and sinistral individuals are frequently found in the flat-shelled stylommatophoran snail genus *Trichocathaica*. We found that three morphologically distinguishable species had been classified as *T.amphidroma*, and thus, treated as three species including *T.macrosquamata* sp. n. and *T.vestita*. We recognized them based on the traits of their shell surface morphology.

In flat-shelled stylommatophoran snail groups, which ordinarily mate simultaneously reciprocally, it is unusually frequent to find both dextral and sinistral specimens in five out of the nine specimen lots. Observations of three cases of enantiomorphism across the three congeneric species are also extraordinary in flat snails. These numerical data rule out the possibility of transient enantiomorphism that results from stochastic appearance of the reversed morph in a simultaneously reciprocally mating population.

Transient enantiomorphism maybe recurrent, however, if snails of *Trichocathaica* employ non-reciprocal copulation by shell mounting. For example, snails of the genus *Oreohelix* of the family Oreohelicidae copulate non-reciprocally, exceptionally for flat groups (Webb 1951). It has been noted that sinistral variants are more frequently found in this group than in other genera in North America (Pilsbry 1939). In the present cases, enantiomorphs were collected in each of the three species only once in 1880s, 1930 or 2015. Thus, transient enantiomorphism in non-reciprocally mating populations might also be the case in the present genus. Our results, therefore, indicate the importance of explicit examination of their copulation mode and quantitative surveys of chiral variation in this genus.

In the Camaenidae, tree snails of the subgenus Amphidromus present exceptions regarding the mating mode and interchiral copulation. They are tall-shelled snails but mate simultaneously reciprocally (Sutcharit et al. 2007; Schilthuizen et al. 2007). Despite their mating mode, they frequently achieve interchiral copulation. Their populations exhibit enantiomorphism, probably because interchiral copulation occurs more frequently than expected in random mating (Schilthuizen et al. 2007). If any mechanism were similarly in effect for maintenance of enantiomorphism in the present three species, both dextrals and sinistrals would have been found commonly across the distribution range of each species. However, the dextral and sinistral specimens were present in mixture in only one of lots that included specimens of each species. Thus, the enantiomorphism in *Trichocathaica* could not be ascribed to balanced enantiomorphism which is positively maintained within populations.

The hypothesis of speciation by left-right reversal predicts morphological divergence following the completion of sexual isolation between populations of enantiomorphs. Shell traits do not necessarily diverge even between genetically isolated good species with distinct genital morphology (Kameda and Fukuda 2015). For comparison of the patterns of penial microsculpture in this study, only a single individual specimen was available for each of the enantiomorphs in *T.vestita*. Thus, there was technical limitation to demonstrate difference associated with enantiomorphs instead of individuals, especially if their populations began to diverge recently. We found that these dextral and sinistral specimens slightly differed in penial sculpture pattern, although their differences were not large enough to describe them as morphologically distinct species. Thus, our results do not rule out the possible presence of sexual isolation between these enantiomorphs. We know no study that evaluated individual variation in penial microsculpture. The present study is the first example of comparing the patterns of penial microsculpture between conchologically indistinguishable enantiomorphs that were locally frequent and repeatedly found among closely related flat species. Our results demonstrate the critical importance of further explicit examination of possible interchiral divergence in penial morphology in *Trichocathaica* as well as in the other stylommatophoran snails.

## Supplementary Material

XML Treatment for Camaenidae

XML Treatment for Trichocathaica

XML Treatment for Trichocathaica amphidroma

XML Treatment for Trichocathaica macrosquamata

XML Treatment for Trichocathaica vestita

XML Treatment for Trichocathaica foliosquama

XML Treatment for Trichocathaica goepeliana

XML Treatment for Trichocathaica lyonsae

XML Treatment for Trichocathaica lyonsae lyonsae

XML Treatment for Trichocathaica lyonsae comosa

XML Treatment for Trichocathaica puteolata

XML Treatment for Trichocathaica rugosobasis

XML Treatment for Trichocathaica
